# Spleen-Derived CCL9 Recruits MDSC to Facilitate Tumor Growth in Orthotopic Hepatoma Mice

**DOI:** 10.1055/s-0043-1777327

**Published:** 2023-12-01

**Authors:** Baohua Li, Wenjuan Li, Yingxue Liang, Chen Zhang, Guangyao Kong, Zongfang Li

**Affiliations:** 1 General Surgery Department of Cadre's Ward, The Second Affiliated Hospital, Xi'an Jiaotong University, Xi'an, People's Republic of China; 2 National & Local Joint Engineering Research Center of Biodiagnostics and Biotherapy, The Second Affiliated Hospital, Xi'an Jiaotong University, Xi'an, People's Republic of China; 3 Core Research Laboratory, The Second Affiliated Hospital, Xi'an Jiaotong University, Xi'an, People's Republic of China; 4 Tumor Immunology Center of Precision Medical Research Institute, The Second Affiliated Hospital, Xi'an Jiaotong University, Xi'an, People's Republic of China

**Keywords:** spleen, CCL9, MDSC, hepatocellular carcinoma

## Abstract

**Objectives** Spleen is involved in multiple diseases, the role of the spleen and spleen-derived factors in hepatocellular carcinoma (HCC) is still not clarified.

**Methods** In the current study, a murine H22 orthotopic hepatoma model was established. Three groups were divided: normal mice, tumor-bearing mice with spleen-preserving, and tumor-bearing mice with splenectomy. Spleen and tumor weights were recorded by weeks 1 and 2. The proportion of myeloid-derived suppressor cell (MDSC) in peripheral blood and tumor tissue was detected using flow cytometry. Protein chip assay was used to compare the differential cytokines between normal liver supernatant and tumor supernatant. The common upregulated cytokines both in spleen and tumor were focused and analyzed using gene expression profiling interactive analysis (GEPIA) database. Enzyme-linked immunosorbent assay was performed to verify the chip result, and to examine CCL9 expression before and after splenectomy. Spleen MDSC was sorted using flow cytometry, and chemotaxis assay was performed to demonstrate whether CCL9 attracted spleen MDSC.

**Results** The spleen enlarged during tumor progression, and compared with splenectomy group, there were faster tumor growth, shorter survival time, and higher proportions of MDSC in spleen-preserving group. Protein chip assay and GEPIA database revealed CCL9 was the most promising chemokine involved in HCC upregulated both in spleen and tumor tissue. CCL9 attracted MDSC in vitro, the level of CCL9 in tumor tissue was downregulated, and the percentage of MDSC was decreased after splenectomy.

**Conclusion** The results demonstrate that CCL9 may be derived from spleen; it facilitated HCC growth via the chemotaxis of MDSC, targeting CCL9 may be a promising strategy in HCC treatment.

## Introduction

Hepatocellular carcinoma (HCC) is the primary malignant tumor in the liver, and it is the third cause of cancer-related death in the world. 1 The treatments of HCC include liver transplantation, percutaneous radiofrequency ablation, transcatheter arterial chemoembolization, sorafenib, immunotherapy based on immune checkpoint blockade targeted on CTLA-4 or PD-1, immunotherapy based on cytokine-induced killer cell, as well as anti–vascular endothelial growth factor antibody. 1 More than half of HCC patients do not respond well to systemic treatment, and normal liver tissues are seriously damaged. 2 3 Therefore, it is of vital importance to elucidate the mechanism of HCC.

Spleen is the largest peripheral immune organ, and many T cells, B cells, macrophages, dendritic cells (DCs), and natural killer T (NKT) cells are in it. The natural and acquired immune responses can be induced in spleen. Spleen is involved in many diseases, including thrombosis, diabetes, lipid metabolism, malaria, acute kidney injury, sepsis, ischemic cardiomyopathy, and neuron immune disease. 4 5 6 7 8 9

The role of spleen in tumor progression is complex and uncertain. Tuftsin is a spleen-derived peptide, exhibiting obvious antitumor effect. 10 Spleen NK cells are the first defense of immune response, and they can kill mutant tumor cells. 11 Splenectomy decreased the percentage of T cells and promoted the growth of W256 tumor. 12 Splenectomy on day 4 in mice with colon cancer increased the expression of Foxp3 in liver, thus facilitated liver metastasis. 13 Liver tumor was transplanted orthotopically, after simultaneous splenectomy, the activity of NK cells was decreased, the number of pulmonary metastases was more and the survival rate was lower in splenectomy group. 14 The recurrence of gastric cancer and pancreatic cancer was inhibited, and the survival time was prolonged in spleen-reserved patients. 15 16

Spleen also has immune-inhibitory effect in tumor immunity. In gastric cancer patients, CD62L positive cells migrated to spleen and produced transforming growth factor (TGF)-β, thus induced the generation of regulatory T cells (Tregs), therefore inhibited immune response. 15 In gene-modified spontaneous pancreatic cancer mice, immunoinhibitory cells including Tregs, myeloid-derived suppressor cells (MDSCs), and tumor-associated macrophages (TAMs) predominated from the early immune response. 17 Spleen CD11b ^+^ Gr-1 ^int^ Ly6C ^hi^ cells closely touched with memory CD8 ^+^ T cells, which made CD8 ^+^ T cells tolerant. Splenectomy as well as immunotherapy restored the function of lymphocytes and promoted tumor regression. 16 It is reported that spleen is the reservoir of monocyte and granulocyte precursors. These precursors were mobilized from spleen to tumor stroma and differentiated into TAMs and tumor-associated neutrophils (TANs). Splenectomy before or after tumor occurrence significantly decreased TAMs and TANs response, and retarded the growth of lung adenocarcinoma. 18

The percentages and activities of TAMs, TANs, Tregs, and MDSCs were increased in peripheral blood and tumor tissue of HCC patients, while the activities of NK and NKT cells were declined. 3 19 Meanwhile, the percentages of total T cells and CD4 ^+^ T cells, as well as the activity of NK cells were all decreased in spleen venous blood of HCC patients, indicating the immune system of HCC patients is impaired, and the immune response of spleen is inhibited.

Therefore, how the spleen affects tumor progression needs to be clarified. In the current study, we established a murine H22 orthotopic hepatoma model, and found that spleen enlarged and promoted hepatoma growth, and facilitated the accumulation of MDSCs in peripheral blood and tumor tissue. Then, we explored what factors in the spleen influenced tumor growth. We focused on spleen-derived cytokines, and found CCL9 was upregulated both in the spleen and tumor tissue. CCL9 could attract MDSCs to tumor tissue. The level of CCL9 in tumor tissue was decreased after splenectomy. So, spleen-derived CCL9 promoted tumor growth via the chemotaxis of MDSC in murine orthotopic hepatoma mice.

## Materials and Methods

### Cell Culture

The murine hepatoma cell lines H22 and Hepa1–6 were purchased from the China Center for Type Culture Collection (Wuhan, China). Cells were cultured in RPMI 1640 medium (HyClone; GE Healthcare, Little Chalfont, UK) and Dulbecco's modified Eagle's medium (HyClone) containing 10% fetal calf serum (HyClone) and 1% penicillin/streptomycin (HyClone) at 37°C in culture chamber with 5% CO _2_ , respectively.

### Mice

Female BALB/c mice (6–8 weeks old) purchased from the animal center of Xi'an Jiaotong University were housed under specific pathogen-free conditions. All experimental manipulations were undertaken in accordance with the National Institutes of Health Guide for the Care and Use of Laboratory Animals, with the approval of the Xi'an Jiaotong University Animal Care and Use Committee (Xi'an, China).

### Orthotopically Implanted Hepatoma Model

Mice were injected 0.1 mL of H22 cells intraperitoneally; 7 days later, the ascites were collected and washed twice with phosphate buffer saline, then adjusted to a concentration to 2 × 10 ^6^ /mL, 20 μL of ascites was injected under the capsule of the left lobe of liver. The orthotopically implanted hepatoma model was established. The tumor-bearing mice were divided into two groups: spleen-preserving group (TB) and splenectomy group (TB + spx). Splenectomy was performed at the meantime of tumor inoculation.

### Flow Cytometry

The percentage of MDSCs in peripheral blood and tumor tissues was analyzed after red blood cells lysis. Red blood cells were lysed using ammonium chloride-potassium lysis buffer (0.15 mol/L NH _4_ Cl, 1 mmol/L KHCO _3_ , and 0.1 mmol/L EDTA, pH 7.2). The cells (10 ^6^ ) were first blocked with anti-CD16/CD32 antibodies for 10 minutes at 4°C, then stained with FITC anti-Ly-6G/Ly-6C (Gr-1) (clone RB6–8C5) (BioLegend, San Diego, California, United States), PE anti-CD11b (clone M1/70) (eBioscience, San Diego, California, United States), and PerCP anti-CD45 monoclonal antibodies (BioLegend) for 30 minutes at 4°C. The stained cells were washed twice and detected by flow cytometry (Canto II, BD BioSciences, United States). The data were analyzed using Diva 7.0 and FlowJo V.7.6.1 software (Tree Star, Inc.).

### Cytokine Array

The expression of cytokines in the liver tissue of normal mice and hepatoma tissue of tumor-bearing mice was examined using RayBiotech mouse cytokine antibody array G-series 3 (catalog No. AAM-CYT-G3–4) according to the established protocol. The chip contains antibodies for 62 cytokines. In brief, the chip was blocked, incubated, and washed, then was scanned with an Axon GenePix scanner (GenePix 4000B, Axon Instruments, United States). The data were annotated and processed with GenePix Pro 6.0 software, and the results were normalized using the positive controls.

### Enzyme-Linked Immunosorbent Assay

The expression of CCL9 in the spleens of normal mice and hepatoma mice, and in the supernatants of normal liver tissue and hepatoma tissue was detected using murine enzyme-linked immunosorbent assay kits (Westang, Shanghai, China) according to the manufacturer's protocols. Each sample, blank, and standard was assayed in duplicate.

### Quantitative Real-Time Polymerase Chain Reaction

Total RNAs were extracted from Hepa1–6 and H22 cell lines using Trizol reagent (Thermo Fisher Scientific, United States). The first-strand complementary DNA was generated using a PrimeScript RT Master Mix (Takara Bio Inc., Japan), and then quantitative real-time polymerase chain reaction (qRT-PCR) was conducted by TB Green Premix Ex *Taq* II (TaKaRa) using GAPDH as the endogenous control. The forward primer for CCL9 was 5′-TCCAGAGCAGTCTGAAGGCACA-3′, and the reverse primer was 5′-CCGTGAGTTATAGGACAGGCAG-3′. The forward primer for GAPDH was 5′-CATCACTGCCACCCAGAAGACTG-3′, and the reverse primer was 5′-ATGCCAGTGAGCTTCCCGTTCAG-3′. qRT-PCR was measured by an ABI 7500 fast real-time PCR system (Applied Biosystems, Foster City, California, United States) and performed in triplicate independently.

### Chemotaxis Assay

In vitro chemotaxis of MDSC was evaluated in 24-well Transwell plates (pore size = 8 μm; Corning); 100 μL of splenic MDSC (10 ^5^ ) in serum-free RPMI 1640 (HyClone) was plated in the upper chamber, and 500 μL of recombinant murine CCL9 (0, 10 ng/mL) (BioLegend) was added to the lower chamber. The Transwell plates were incubated at 37°C, 5% CO _2_ for 3 hours. The migrated MDSCs in the lower chamber were stained with crystal violet, and the numbers were counted under microscopy.

### Statistical Analysis

All data were presented as the mean ± standard deviation. The statistical significance between the two groups was determined by Student's *t* -test. The relationship between spleen weight and tumor weight was assessed by Pearson's correlation. Linear regression analysis was used to confirm the association. Statistical calculations were conducted using PRISM 8 software (GraphPad Software Inc., La Jolla, California, United States). A *p* < 0.05 was considered statistically significant.

## Results

### Spleen Promoted Tumor Growth in H22 Orthotopic Hepatoma Mice

**Fig. 1:**
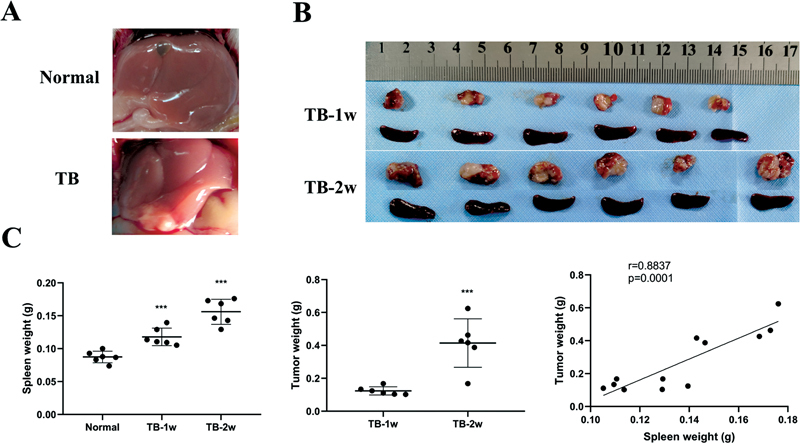
Spleen enlarged during tumor growth in orthotopic hepatoma mice. (A) Normal liver and hepatoma tissue. (B) The images of tumor and spleen by weeks 1 and 2 ( *n* = 6). (C) Spleen weight (*** *p* < 0.001 compared with normal mice), tumor weight (*** *p* < 0.001 compared between weeks 1 and 2), and their correlation ( *p* = 0.0001). TB, tumor-bearing.

Large amounts of ascites were formed on day 7 after the injection of H22 cell line into the abdominal cavity. The ascites were injected into the left lobe of the liver to establish a murine orthotopic hepatoma model ( Fig. 1A ). The spleen weight and tumor weight were measured by weeks 1 and 2. The correlation between them was also analyzed. The spleen enlarged during tumor growth, and the spleen weight was positively correlated with tumor weight ( Fig. 1B, C ).

**Fig. 2:**
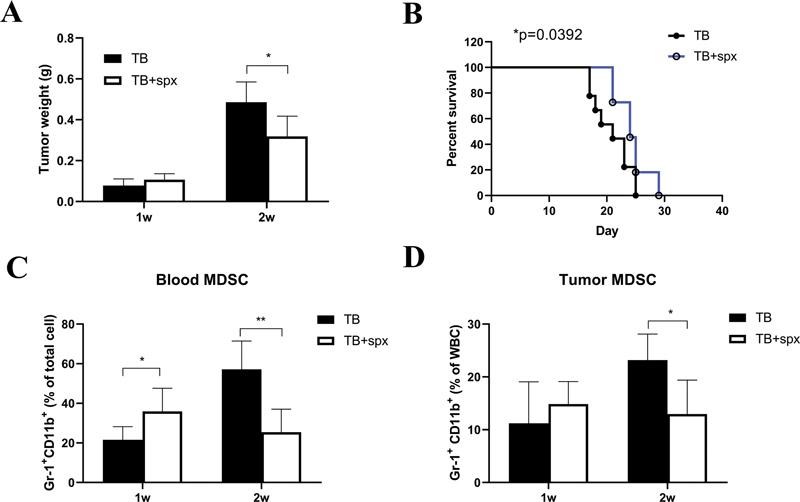
Spleen promoted tumor growth and the accumulation of MDSCs. (A) The changes of tumor weight after splenectomy by weeks 1 and 2 in orthotopic hepatoma mice ( *n* = 5). (B) The survival curve of hepatoma mice before and after splenectomy ( *n* = 10). The alteration of the percentage of MDSC in peripheral blood (C) and tumor tissue (D) after splenectomy in hepatoma mice ( *n* = 6). MDSCs, myeloid-derived suppressor cells; Spx, splenectomy; TB, tumor-bearing. * *p* < 0.05, *** *p* < 0.001.

We performed splenectomy just before tumor cell inoculation, tumor weight was decreased by week 2 ( Fig. 2A ), and the survival of tumor-bearing mice was prolonged after splenectomy (24.36 ± 2.803 vs. 20.89 ± 3.257 days, *p* = 0.0392) ( Fig. 2B ). The percentage of MDSCs in peripheral blood ( Fig. 2C ) and tumor tissue ( Fig. 2D ) was decreased by week 2 after splenectomy. These results indicate spleen may promote the accumulation of MDSCs both in peripheral blood and tumor tissue, and thus facilitate tumor growth.

### CCL9 Was Highly Expressed Both in Spleen and Tumor Tissue of Hepatoma Mice

**Fig. 3:**
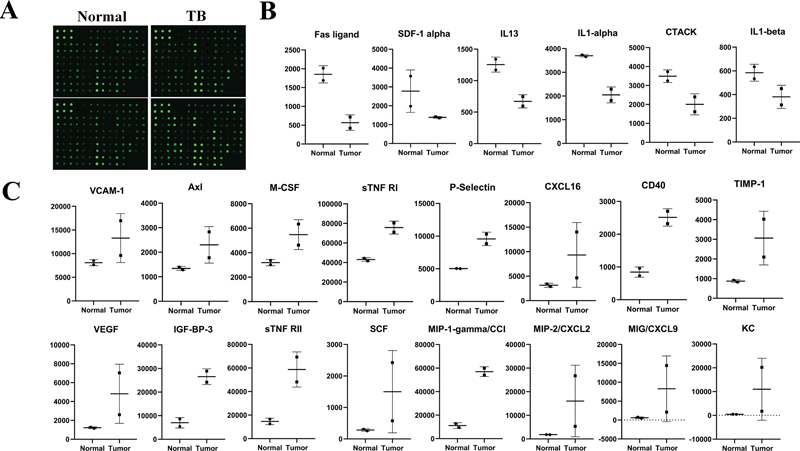
Differential expression of 62 cytokines in the supernatants of normal liver and tumor tissue. (A) Mouse cytokine antibody array membranes. (B) The downregulated cytokines and (C) the upregulated cytokines expressed in tumor supernatant of hepatoma mice. TB, tumor-bearing.

**Fig. 4:**
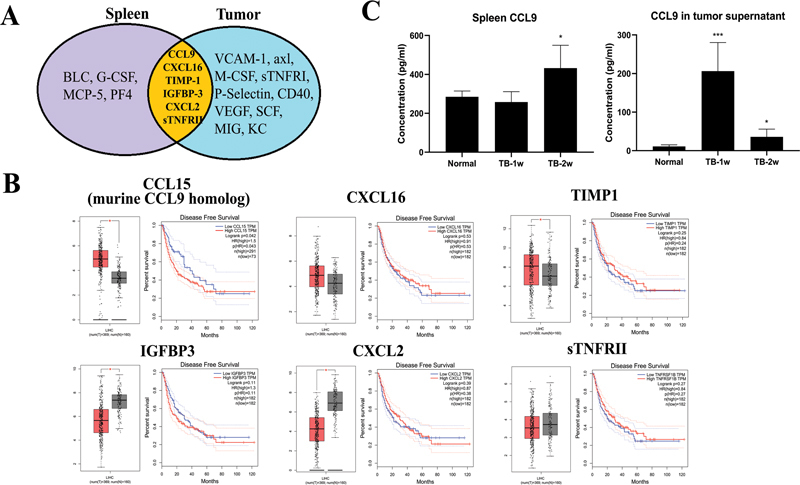
CCL9 was upregulated both in spleen and tumor tissue of hepatoma mice. (A) The six common upregulated cytokines both expressed in spleen and tumor tissue of hepatoma mice. (B) Six common upregulation cytokines in human HCC from GEPIA database. (C) The verification of the expression of CCL9 in spleen and tumor tissue in normal mice and TB mice ( *n* = 6) by ELISA. * *p* < 0.05, *** *p* < 0.001 compared with normal mice. ELISA, enzyme-linked immunosorbent assay; TB, tumor-bearing.

To clarify what factors in the spleen influenced tumor growth, the upregulated cytokines both in spleen and tumor tissue were focused. The protein chip assay ( Fig. 3A ) showed that compared with normal liver tissue, there were 6 cytokines downregulated (≥1.5) ( Fig. 3B , Table 1 ) and 16 cytokines upregulated (≤0.65) ( Fig. 3C , Table 2 ) in tumor tissue. Among the upregulated cytokines, the levels of CCL9, CXCL16, TIMP-1, IGF-BP-3, CXCL2, and sTNFRII were elevated both in spleen and tumor tissue ( Fig. 4A ) (the spleen chip was previously published). 20 GEPIA database showed that CCL15 (human ortholog of mouse CCL9), CXCL16, and TIMP-1 were increased in HCC patients, whereas IGF-BP-3, CXCL2, and sTNFRII were decreased in HCC patients ( Fig. 4B ). The survival curve showed that higher CCL9 had poorer survival, while the survival curve of CXCL16 and TIMP-1 had no significant difference ( Fig. 4B ). So, CCL9 was probably one of the most important cytokines in HCC. It was verified that the level of CCL9 was upregulated in the spleen and tumor tissue of hepatoma mice ( Fig. 4C ).

**Table 1:** Downregulated cytokines in hepatoma tissue compared with the liver tissue of normal mice

Protein ID	Full name	Fold change
FAS ligand	Fas ligand	0.30
SDF-1 α	Stromal cell-derived factor-1 α	0.52
IL-13	Interleukin-13	0.53
IL-1-α	Interleukin-1 α	0.55
CTACK	Cutaneous T cell-attracting chemokine	0.56
IL-1-β	Interleukin-1 β	0.64

**Table 2:** Upregulated cytokines in hepatoma tissue compared with the liver tissue of normal mice

Protein ID	Full name	Fold change
VCAM-1	Vascular cell-adhesion molecule 1	1.58
axl	AXL receptor tyrosine kinase	1.67
M-CSF	Macrophage colony-stimulating factor 1	1.70
sTNF RI	Soluble tumor necrosis factor receptor type I	1.76
P-selectin	P-selectin	1.89
CXCL16	CXC-chemokine ligand 16	2.57
CD40	CD40	2.99
TIMP-1	Tissue inhibitor of metalloproteinases 1	3.33
VEGF	Vascular endothelial growth factor	3.49
IGF-BP-3	Insulin-like growth factor binding protein-3	3.87
sTNF RII	Soluble tumor necrosis factor receptor type II	3.99
SCF	Stem cell factor	4.18
MIP-1-gamma/CCL9	Macrophage inflammatory protein 1 gamma	5.17
MIP-2/CXCL2	Macrophage inflammatory protein 2	6.50
MIG/CXCL9	Monokine induced by interferon-γ	9.18
KC	Keratinocyte-derived chemokine	13.24

### CCL9 May Be Derived from Spleen

**Fig. 5:**
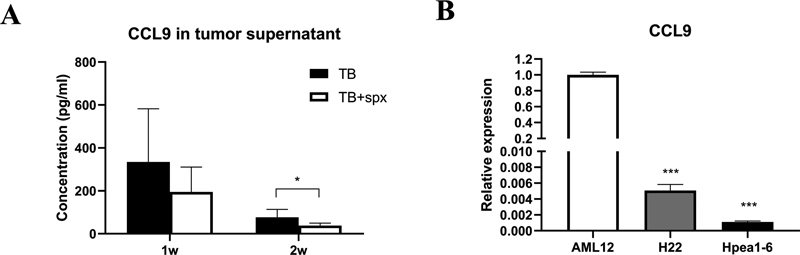
The upregulated tumor CCL9 was derived from the spleen. (A) The alteration of the level of tumor CCL9 after splenectomy in H22 orthotopic hepatoma mice ( *n* = 6) detected by ELISA. (B) The relative expression of CCL9 in normal liver cell line (AML12) and liver tumor cell lines (H22 and Hepa1–6) detected by qRT-PCR. * *p* < 0.05 and *** *p* < 0.001. ELISA, enzyme-linked immunosorbent assay; qRT-PCR, quantitative real-time polymerase chain reaction.

It was shown that the expression of CCL9 was decreased in tumor tissue after splenectomy in tumor-bearing mice ( Fig. 5A ), indicating that the spleen may affect the expression of CCL9 in tumor tissue. As CCL9 was also upregulated in the spleen, CCL9 in tumor tissue was perhaps derived from the spleen. We wondered if hepatoma cell lines could produce high amounts of CCL9, then the level of CCL9 in normal liver cell line (AML12) and hepatoma cell lines (H22 and Hepa1–6) was compared. It was found that the level of CCL9 in H22 and Hepa1–6 was lower than normal liver cell line AML12 ( Fig. 5B ), suggesting that the high level of CCL9 in tumor tissue is not derived from tumor cell lines, further indicating that CCL9 may be derived from the spleen.

### CCL9 Promoted Tumor Growth via Recruiting MDSCs to Tumor Tissue

**Fig. 6:**
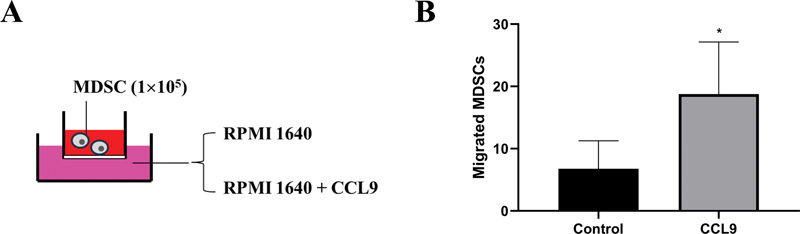
CCL9 could attract spleen MDSCs. (A) MDSCs were sorted from tumor-bearing mice by flow cytometry, and were seeded in the upper chamber of 8-μm Transwell plates. Murine recombinant CCL9 (10 ng/mL) or RPMI 1640 medium was added in the lower chamber. (B) Three hours later, MDSCs in the lower chamber were stained with crystal violet, and the number of MDSCs was counted under ×200 microscopy. * *p* < 0.05. MDSCs, myeloid-derived suppressor cells.

Since the percentage of MDSCs was decreased and the level of CCL9 was lowered in tumor tissue after splenectomy, whether CCL9 played a role in MDSC accumulation in tumor tissue, chemotaxis assay was performed and showed that CCL9 attracted more spleen MDSCs than control medium in vitro ( Fig. 6 ), indicating spleen MDSCs may be migrated to tumor tissue via the chemotaxis of CCL9 highly expressed in tumor.

**Fig. 7:**
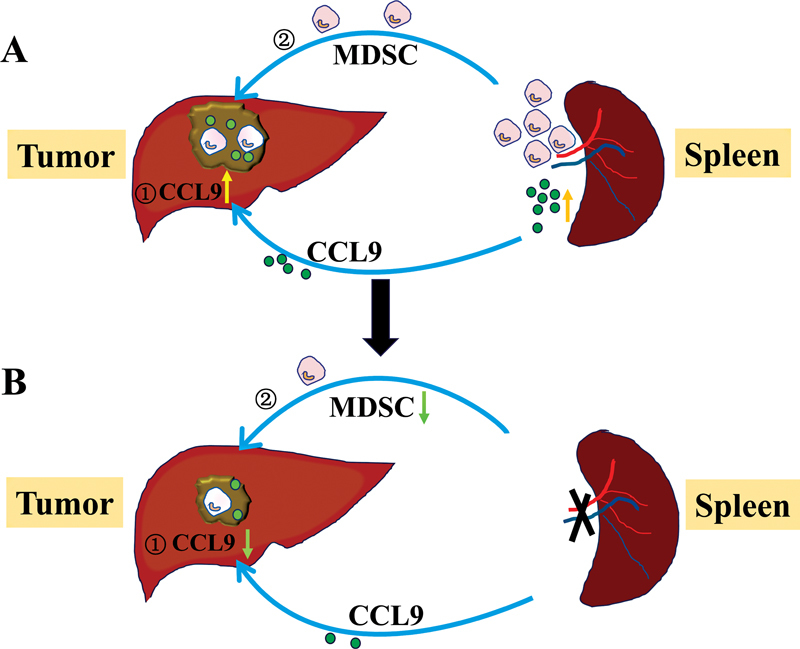
Schematic diagram of spleen-derived CCL9 promoted tumor growth via the accumulation of MDSCs. (A) (1) Spleen-derived CCL9 was secreted into blood and entered tumor tissue, (2) then attracted spleen MDSC to liver tumor, thus promoted tumor growth. (B) (1) The downregulation of CCL9 after splenectomy could (2) decrease the percentage of MDSC and inhibited tumor growth. MDSCs, myeloid-derived suppressor cells.

Taken together, in murine H22 orthotopic hepatoma model, spleen produced high level of CCL9, CCL9 circulated in blood; therefore, it was also elevated in tumor tissue, and attracted spleen MDSCs to hepatoma tissue, thus facilitated tumor growth ( Fig. 7A ). After the removal of the spleen, the expression of CCL9 was downregulated and the percentage of MDSCs was decreased, thus tumor growth was inhibited ( Fig. 7B ). CCL9 may be a promising target in HCC therapy.

## Discussion

In the current study, it was found that spleen promoted the accumulation of MDSCs in peripheral blood and tumor tissue, and thus facilitated tumor growth via CCL9. There are several literatures also reported the role of spleen in tumor progression via other mechanisms. Wu et al reported that in solid tumors, spleen exhibited heightened extramedullary hematopoiesis, splenic early hematopoietic stem/progenitor cells were committed to generate MDSCs, thus promoted tumor growth, and the abrogation of splenic myelopoiesis showed promising effects in enhancing therapeutic antitumor immune responses. 21 22 23 Levy et al reported that in several murine lung cancer model, spleen was an important contributor to tumor growth and metastases substantially mediated by MDSCs. Splenectomy significantly reduced the presence of MDSCs in circulation and inside the tumor. 24 So, spleen plays an important role in tumor progression, specifically targeting spleen-derived factors may be a promising strategy in tumor therapy.

MDSCs are a heterogenous population of immature myeloid cells that have potent immune-suppressive activity. In both mice and humans, MDSCs accumulate in most individuals with cancer, and they inhibit antitumor immunity and promote tumor growth. MDSCs have several chemokine receptors, including CCR1, CCR2, CCR3, CCR5, CCR12, CXCR2, CXCR4, and C5aR1. 25 In the current study, MDSCs may express CCR1, the receptor of CCL9, which mediated the migration of MDSCs to tumor tissue.

Spleen-derived cells and cytokines can be mobilized to the pathological tissue. In ischemic heart disease, monocytes were mobilized from the spleen to ischemic myocardium and repaired the wound. 26 In lung adenocarcinoma, spleen-derived TAM and TAN precursors migrated to tumor stroma, and promoted tumor growth. 18 27 In the treatment of type 2 diabetes with adipose tissue-derived stem cells (ADSCs), spleen-derived interleukin 10 (IL-10) was elevated. After splenectomy, IL-10 was decreased, and hyperglycemia as well as insulin resistance was exacerbated, and the effect of ADSCs was attenuated. 28 In endotoxemia, spleen-derived interferon (IFN)-γ induced the generation of suppressive neutrophils, splenectomy nullified circulating IFN-γ level and reduced the percentage of PD-L1 ^+^ neutrophils, ameliorated immune response. 29 In our current study, spleen-derived CCL9 also played important role in tumor-promoting effect via attracting MDSCs to tumor tissue. Further experiments should be performed, including the injection of anti-CCR1 antibody, or the addition of CCL9 after splenectomy, to further confirm the effect of CCL9 in tumor immunity.

CCL9, also known as macrophage inflammatory protein-1 gamma, is often produced in macrophages, osteoclasts, DCs, Langerhan's cells, and functions as a cell survival factor. 30 31 32 CCL9 can also be produced by MDSCs 33 and even by tumor cells. 34 In our current study, we only demonstrate CCL9 may be derived from spleen, but we do not know the cell source of CCL9. Is CCL9 secreted by spleen macrophage, or spleen MDSC, or other cells? It needs further exploration.

CCR1 is the only receptor of CCL9. CCL9/CCR1 signaling has been shown to be important for the recruitment of myeloid progenitors to intestinal tumors, leading to enhanced invasion. 34 TGF-β signaling pathway can activate MDSC to produce CCL9, 35 Baoyuan Jiedu decoction, a traditional Chinese medicine formula, can inhibit the accumulation of MDSC in premetastatic niche of lung via the downregulated TGF-β/CCL9 pathway in 4T1 tumor-bearing mice. 36 So, what is the upstream signaling of CCL9 in H22 orthotopic hepatoma mice? Whether blocking the upstream signaling of CCL9 can inhibit hepatoma growth?

## Conclusion

In summary, in H22 orthotopic hepatoma mice, spleen-derived CCL9 induced MDSC accumulation in the tumor and promoted tumor growth. The downregulation of CCL9 after splenectomy decreased the percentage of MDSC in peripheral blood and tumor, and thus inhibited tumor growth. Targeting CCL9 may be a promising strategy in HCC treatment.
